# Neurosarcoidosis: A Unique Presentation of a Rare Disease

**DOI:** 10.7759/cureus.48499

**Published:** 2023-11-08

**Authors:** Kyrillos Girgis, Danielle Retcho, Raymond Pesenti, Desmond Aroke, Rafail Beshai

**Affiliations:** 1 Internal Medicine, Newark Beth Israel Medical Center, Newark, USA; 2 Cardiovascular Diseases, Virtua, Camden, USA; 3 Internal Medicine, Jefferson Health, Stratford, USA

**Keywords:** rare presentation, rare disease, neurological deficits, neurosarcoidosis, sarcoidosis

## Abstract

Sarcoidosis is defined as an immune-mediated multi-organ granulomatous disease with unknown etiology, which is characterized by the presence of multiple non-caseating granulomas in the absence of a definite infective or toxic cause. Neurosarcoidosis (NS) occurs when sarcoid granulomas invade the central or peripheral nervous systems. Sarcoidosis usually presents with non-specific manifestations, including dry cough, fatigue, night sweats, weight loss, skin changes, and eye manifestations. Many patients who develop NS present with neurological manifestations within two years of being diagnosed with sarcoidosis. Herein, we present a case of newly diagnosed sarcoidosis in a 49-year-old male patient initially presenting with neurological manifestations of unknown origin, later identified as NS on peripheral lymph node biopsy with non-caseating granuloma.

## Introduction

Sarcoidosis is defined as an immune-mediated multi-organ granulomatous disease with unknown etiology, which is characterized by the presence of multiple non-caseating granulomas in the absence of a definite infective or toxic cause [1]. The incidence of sarcoidosis in the United States ranges from 35 to 80 per 100,000 African Americans and three to 10 per 100,000 Caucasians, with a slight female predominance [2]. The lung is the most affected organ. However, sarcoidosis can also affect other organs, including the skin, eyes, liver, and lymph nodes. Neurosarcoidosis (NS) occurs when sarcoid granulomas invade the central or peripheral nervous systems [2]. It is considered rare and has been identified in about 5-10% of all patients with sarcoidosis [2]. Sarcoidosis usually presents with non-specific manifestations, including dry cough, fatigue, night sweats, weight loss, skin changes, and eye manifestations [3]. Many patients who develop NS present with neurological manifestations within two years of being diagnosed with sarcoidosis [2]. However, there are 10-20% of isolated NS that can be seen in patients without systemic symptoms [2].

## Case presentation

Our patient was a 49-year-old male with a history of coronary artery disease, systemic hypertension, hyperlipidemia, and obesity who presented to the hospital for progressive weakness of the lower extremities. The patient reported that he had abdominal pain and bilateral lower back pain for approximately two to three weeks prior, prompting him to visit an outside hospital. A computed tomography (CT) scan of his abdomen and pelvis demonstrated abdominal lymphadenopathy. The patient was instructed to follow up with his primary care physician for concerns of lymphoma. After discharge from that facility, the patient reported that his legs became weak with numbness and tingling resulting in multiple falls from standing height without loss of consciousness. The patient denied urine or stool incontinence. He also denied recent travel, vaccinations, sick contacts, fever, chest pain, shortness of breath, nausea, vomiting, or diarrhea. The patient did not endorse any significant family history.

In the emergency room, his vitals were within normal limits. The bilateral cranial nerve exam was normal without any ocular motor or visual field deficits. The strength of the bilateral upper extremities and bilateral lower extremities was 5/5 and 3/5, respectively. The neurological reflexes were normal in the upper extremities but completely absent in the lower extremities with decreased sensation to light touch in all lower extremity dermatomes. Labs showed a normal complete blood count and basic metabolic panel with no electrolyte abnormalities. The patient was found to have an elevated erythrocyte sedimentation rate of 60 mm/hr and a C-reactive protein of 0.7 mg/dl. Thyroid-stimulating hormone and vitamin B12 levels were within normal limits. Rapid plasma reagin was negative. The antinuclear antibody test and other autoimmune markers were negative. Table 1 summarizes the patient's lab results.

**Table 1:** Patient's lab results

Lab test	Lab value	Reference range
Complete blood count		
White blood cells	6 x 10^9/L	4-10 x 10^9/L
Hemoglobin	13 g/dL	12.5-16.5 g/dL
Platelets	280 x 10^9/L	150-400 x 10^9/L
Basic metabolic panel		
Sodium	138 mmol/L	135-145 mmol/L
Potassium	4 mmol/L	3.5-5 mmol/L
Chloride	100 mmol/L	95-105 mmol/L
Calcium	10 gm/dL	8.5-10.1 gm/dL
Bicarbonate	26 mmol/L	21-32 mmol/L
Glucose	100 mg/dL	75-105 mg/dL
Blood urea nitrogen	11 mg/dL	7-18 mg/dL
Creatinine	0.75 mg/dL	0.7-1.3 mg/dL
Erythrocyte sedimentation rate	60 mm/hr	<20 mm/hr
C-reactive protein	0.7 mg/dL	<0.3 mg/dL
Thyroid-stimulating hormone	1.7 IU/ml	0.35-3.5 IU/ml

**Figure 1:**
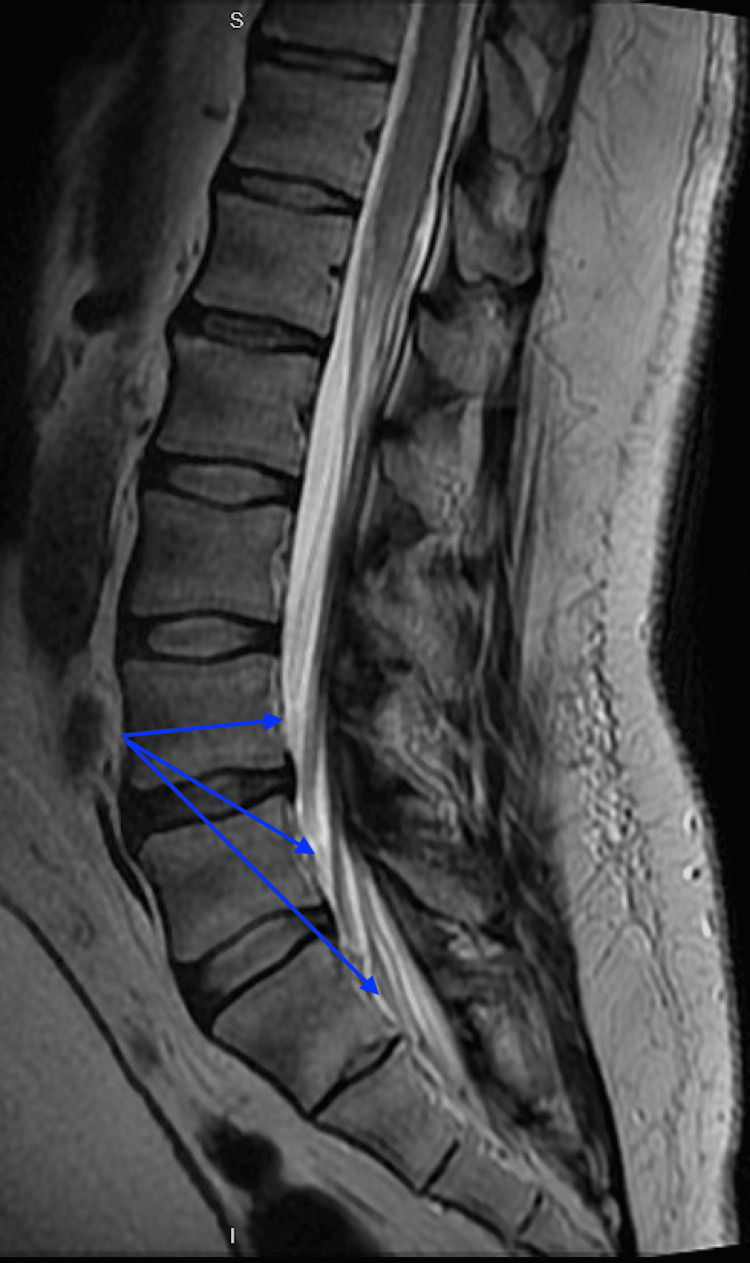
MRI of the lumbar spine showing nodular contiguous ventral and dorsal leptomeningeal enhancement (blue arrows)

Magnetic resonance imaging (MRI) with and without contrast of the lumbar spine (Figure 1) was obtained, demonstrating diffuse nodular contiguous ventral and dorsal leptomeningeal enhancement and nodular enhancement in the central cord with mild increased T2 signal of the conus medullaris and thickening of the nerve roots of the cauda equina. These findings prompted a neurology consultation, who recommended a lumbar puncture. Cerebrospinal fluid was obtained, which demonstrated a slightly elevated WBC count of 40 WBCs/mm3, markedly elevated protein of 600 mg/dl, and decreased glucose of 30 mg/dl. Neurology recommended a trial of intravenous immunoglobulin (IVIG) 40 gm daily in addition to methylprednisolone 1 gm daily for five days. Additionally, general surgery was consulted to obtain a lymph node biopsy for pathological analysis.

**Figure 2:**
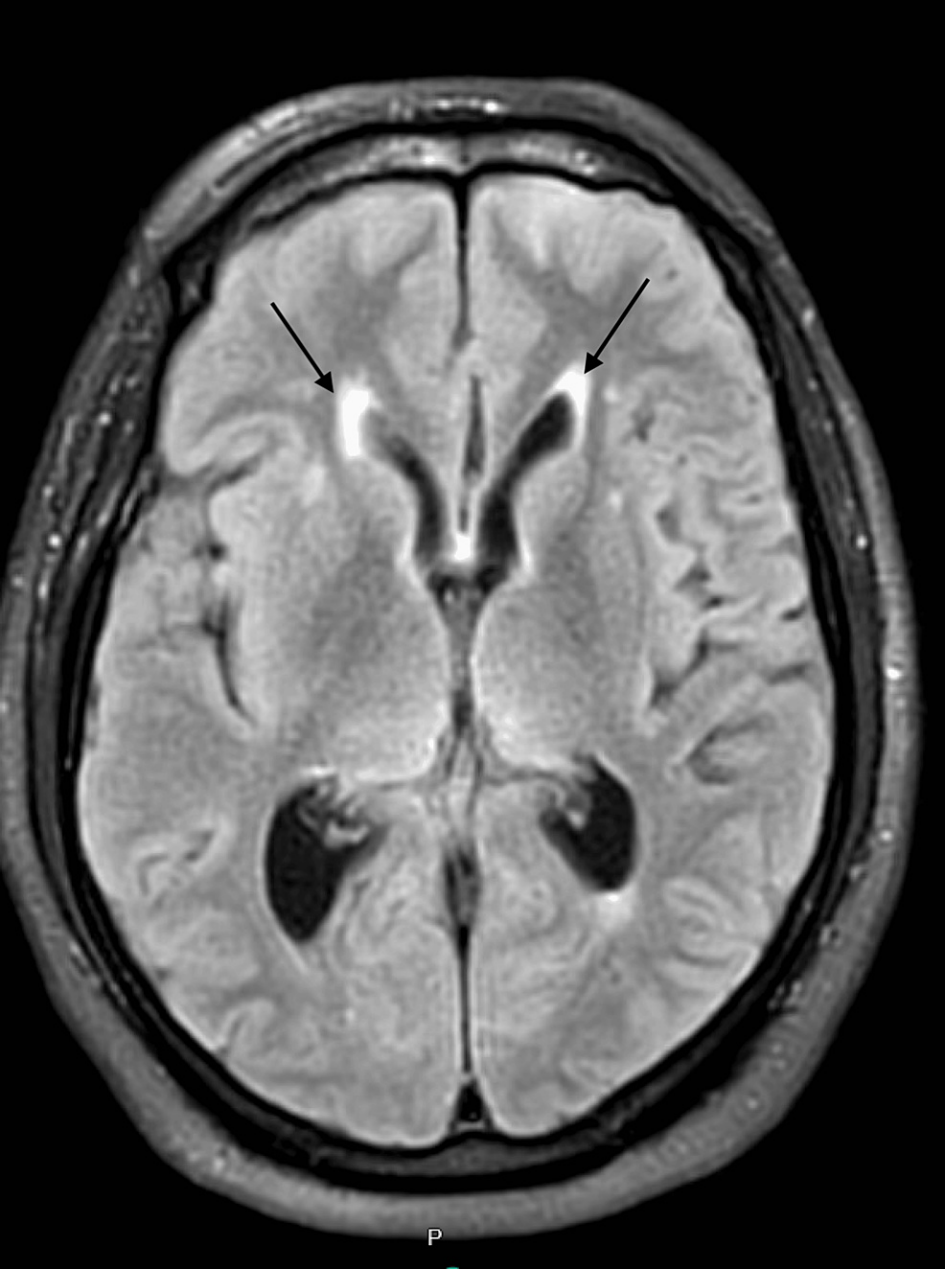
MRI of the brain showing abnormal fluid-attenuated inversion recovery (FLAIR) signals in the periventricular regions (black arrows)

**Figure 3:**
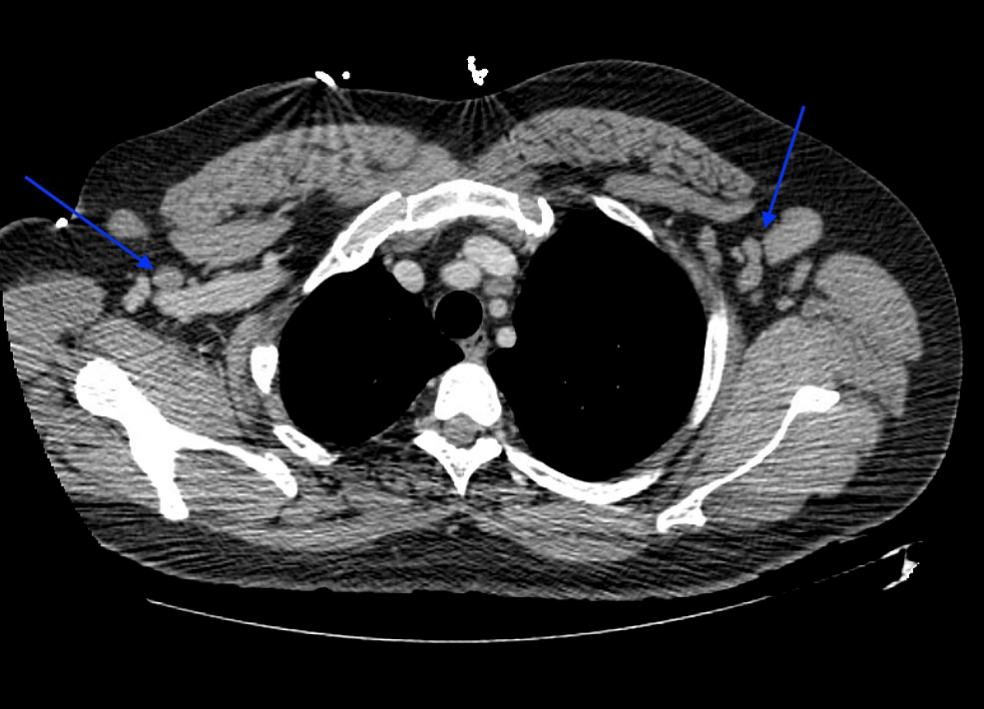
CT of the chest showing bilateral axillary lymphadenopathy (blue arrows)

**Figure 4:**
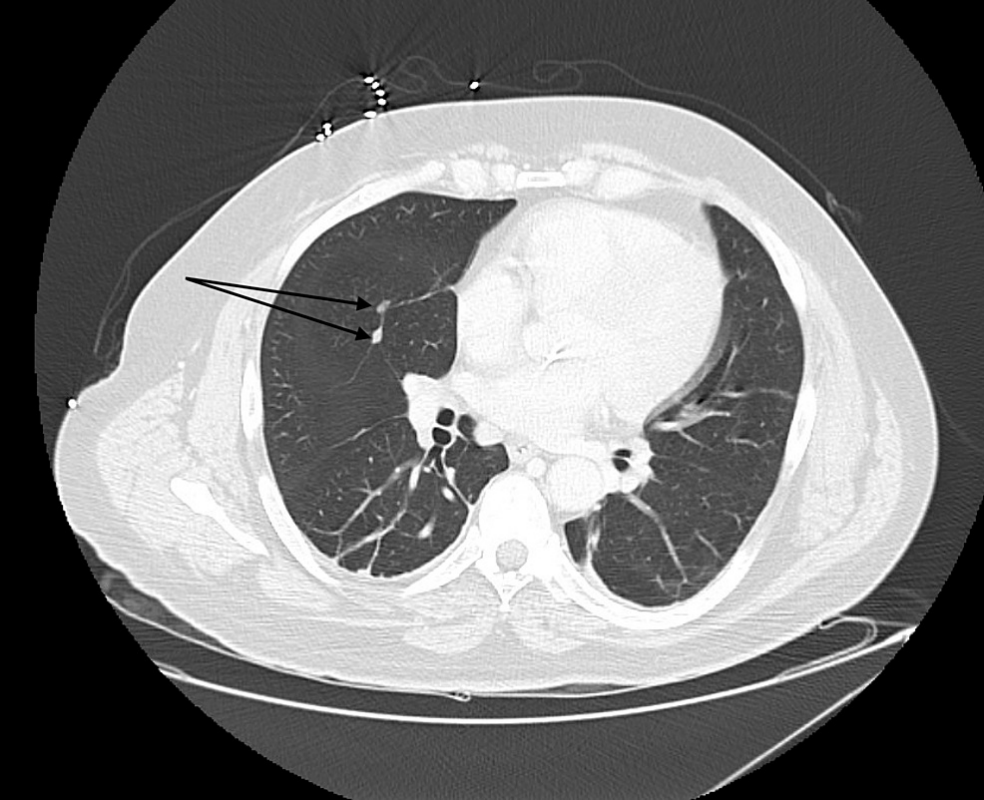
CT of the chest showing two lung nodules in the right middle lobe (black arrows)

The patient underwent further MRI imaging of the cervical and thoracic spine, demonstrating abnormal spinal cord signals suggesting demyelination and transverse myelitis. These findings prompted an MRI of the brain without contrast (Figure 2), which demonstrated abnormal fluid-attenuated inversion recovery (FLAIR) signals in the periventricular regions and along the inferior third ventricle, suggesting atypical demyelination. A pan CT scan demonstrated lymphadenopathy in the axilla (Figure 3), abdomen, and pelvis, in addition to two lung nodules in the right middle lobe (Figure 4), yet no suspicious masses in the abdomen or pelvis were appreciated. At this time, the working diagnoses for the patient included Guillain-Barré syndrome, transverse myelitis, and lymphoma.

**Figure 5:**
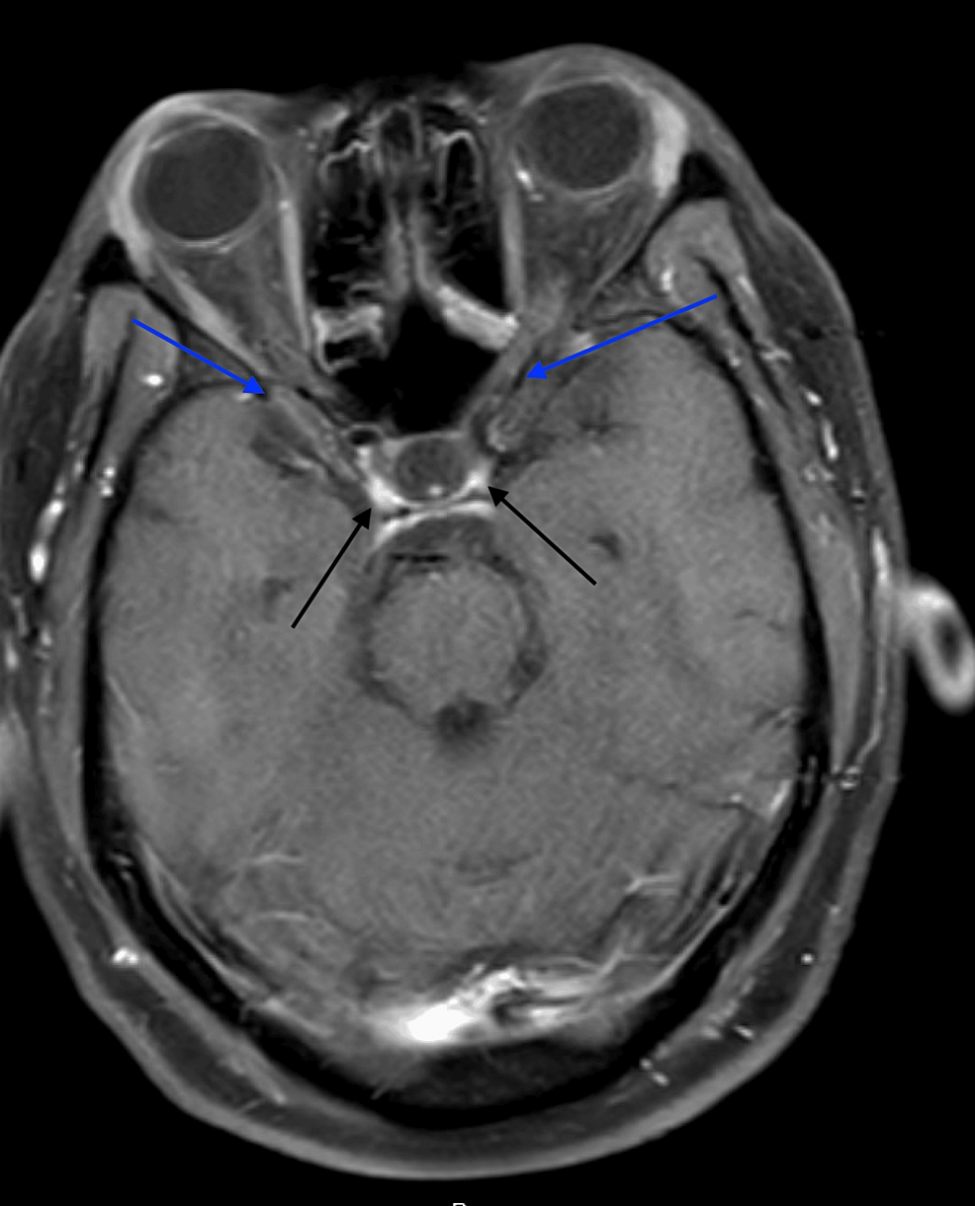
MRI of the brain with contrast showing abnormal enhancement of the posterior portions of the bilateral optic nerves (blue arrows) and optic chiasm (black arrows)

On the third day of admission, he underwent a lymph node biopsy of the right inguinal node. Further imaging after the biopsy included an MRI brain with contrast (Figure 5), which demonstrated abnormal enhancement of the posterior portions of the bilateral optic nerves, hypothalamus, and optic chiasm suggesting neuromyelitis optica spectrum disorder.

The patient received four of the five doses of IVIG and methylprednisolone before he decided to leave the hospital for personal reasons despite continued weakness of his lower extremities bilaterally. The day after his departure, the pathology report revealed non-caseating granulomas and he was informed of the diagnosis of neurosarcoidosis. However, the patient did not follow up.

## Discussion

NS is a rare occurrence in patients with sarcoidosis and often presents with varying neurologic deficits overlapping with conditions such as Guillain-Barré syndrome (GBS) and transverse myelitis. The exact percentage of patients with this disorder varies depending on the papers reviewed, but upwards of 5% of patients with systemic sarcoidosis may develop NS [4]. However, there are 10-20% of isolated NS that can be seen in patients without systemic symptoms of sarcoidosis [2]. A hallmark of sarcoidosis is the formation of granulomas, which interfere with normal tissue architecture and result in organ dysfunction [1,2]. Granulomas consist of CD4+ T helper cells and epithelioid macrophages with a surrounding ring of fibroblasts along with scattered B cells and CD8+ cytotoxic T cells at the edge of the granulomas. Activated macrophages (epithelioid macrophages) produce tumor necrosis factor (TNF)-α, which is important for the development and maintenance of sarcoid granulomas as it stimulates naive CD4+ T cells [2,3]. NS results when sarcoid granulomas invade the central or peripheral nervous system [2,3].

Common central nervous system presentations of NS include leptomeningeal disease, cranial nerve neuropathy, and longitudinally extensive myelitis [2,4]. The peripheral nervous system presentations often overlap with GBS and there may be associated muscle disease due to the granuloma formation, for which electromyography (EMG) and nerve conduction studies might provide some help in distinguishing NS from other neuromuscular disorders [2,4].

Diagnosing NS is very challenging and requires taking into account the patient’s history, physical exam, imaging studies, EMG or nerve conduction studies, CSF findings, and biopsy results [2]. As mentioned previously, there are many neurologic diseases that mimic NS. Therefore, a biopsy showing non-caseating granulomas would be most specific for NS, as seen in our patient. Acute management of NS involves steroids and long-term follow-up with repeat imaging and possible immunologic agents if the condition persists, such as TNF-alpha inhibitors.

Our literature search revealed four other case reports of NS presenting with neurological deficits similar to GBS and transverse myelitis [5-7]. Table 2 summarizes the clinical presentation, imaging, and CSF results as well as pathology findings of these reported cases.

**Table 2:** Literature review

Source	Age (years), gender (M/F)	Presenting symptoms	Physical exam	Imaging findings	CSF	Pathology	
[5]	60, M	Right-sided abdominal pain with paresthesia in the trunk and bilateral lower extremities	Bilateral brisk reflexes, ankle clonus, pyramidal signs, and Babinski sign	MRI spine with C5-T2 hyperintensity; CT chest with multiple enlarged lymph nodes in hilum and mediastinum	Mildly elevated protein (48 mg/dL)	Granulomatous lymphadenitis with non-caseating granulomas	
[6]	47, M	Lower back pain, urinary retention, bilateral lower extremity weakness, and numbness/tingling	Unable to ambulate, brisk reflexes, bilateral lower extremity muscle weakness, sensory loss to T6 dermatome	MRI spine with hyperintensity of cervical and thoracic cord, with patchy enhancement	Lymphocytic pleocytosis and elevated protein	Mediastinal lymph node biopsy with non-caseating granulomas	
[6]	56, M	Right lower extremity followed by right upper extremity weakness and numbness ultimately with hemiparesis (history of respiratory infection 4 weeks prior)	Right-sided hyperreflexia with decreased sensation of right arm and leg (C5-6 pattern)	MRI of the cervical spine with T2 hyperintensity from C2 to C7 with patchy enhancement	Lymphocytic pleocytosis and elevated protein	Mediastinal lymph node biopsy with non-caseating granulomas	
[7]	43, M	Acute worsening limb weakness with sensory changes in hands and feet associated with a prior history of weight loss and vision change	Global areflexia	CT chest with fine nodular pattern in both lungs; MRI of the brain and spine with demyelination	Acellular fluid with mild protein elevation (0.67 g/L)	Bronchoscopy with non-caseating granuloma	

## Conclusions

Patients presenting with neurological abnormalities on physical examination with varying imaging and CSF findings fall into a broad differential. As such, the patient presented here was initially treated with IVIG and a steroid course due to concerns of GBS and transverse myelitis. It was only after a biopsy of the lymph proving non-caseating granulomas without systemic symptoms that neurosarcoidosis was diagnosed.
